# Addressing clinical challenges in ANCA-associated vasculitis with real-world evidence

**DOI:** 10.1093/rheumatology/keaf581

**Published:** 2025-11-03

**Authors:** David Jayne

**Affiliations:** Department of Medicine, University of Cambridge and Addenbrooke’s Hospital, Cambridge, UK

**Keywords:** ANCA, ANCA-associated vasculitis, AAV, real-world studies, glucocorticoids, avacopan

## Abstract

Key challenges in the management of ANCA-associated vasculitis (AAV) include the need to achieve more rapid and sustained remission, reduce exposure to glucocorticoids (GC) and reliably monitor and predict treatment response. Clinical trials in patients receiving rituximab or cyclophosphamide for AAV show that the adjunctive use of avacopan (a novel complement 5a receptor 1 [C5aR1] antagonist) for up to 1 year enables sustained AAV remission, considerable reductions in GC exposure, and greater recovery of kidney function, especially in patients with acute kidney injury. Additional real-world evidence suggests avacopan can be used to replace GC in patients with GC toxicity and supports the use of avacopan in AAV patients with rapidly progressing glomerulonephritis, pulmonary hemorrhage and/or refractory AAV. Future studies are needed to investigate the benefits of extending avacopan treatment beyond 1 year and in specific populations.

## Graphical abstract

**Figure:**
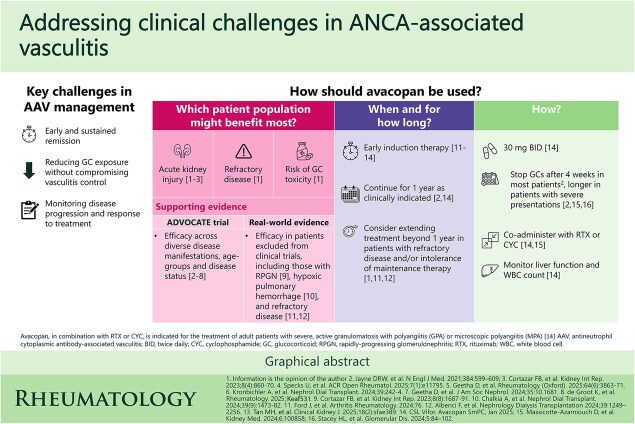

Rheumatology key messages

- Effective ANCA-associated vasculitis (AAV) management requires more rapid and sustained remission and reduced glucocorticoid exposure.
- Avacopan permits sustained AAV remission, reduced glucocorticoid exposure and improved recovery of kidney function.
- Real-world evidence supports the use of avacopan in AAV patient subgroups.

## Introduction

ANCA-associated vasculitis (AAV) is a group of multisystem autoimmune disorders which includes granulomatosis with polyangiitis (GPA), microscopic polyangiitis (MPA) and eosinophilic granulomatosis with polyangiitis (EGPA) [1, 2]. Treatment for AAV is designed to reduce the risk of vasculitis-related organ damage and inflammation-related comorbidities using intensive induction therapy to rapidly control active disease, followed by less-aggressive maintenance therapy to reduce the risk of AAV relapse [3, 4]. According to treatment guidelines, therapies for GPA and MPA should typically include rituximab (RTX) or cyclophosphamide (CYC), alongside glucocorticoids (GC) and/or avacopan [3–5]. Although effective, AAV treatment strategies are associated with a number of challenges. These include the need to achieve more rapid and sustained AAV remission, reduce patient exposure to GC and reliably assess and predict treatment response in different patient subgroups. In this review, we consider ways in which these challenges might be addressed, focusing on the use of avacopan (a novel complement 5a receptor 1 [C5aR1]) in real-world clinical practice.

## Challenges in the treatment of AAV

### AAV remission

**Figure 1.**
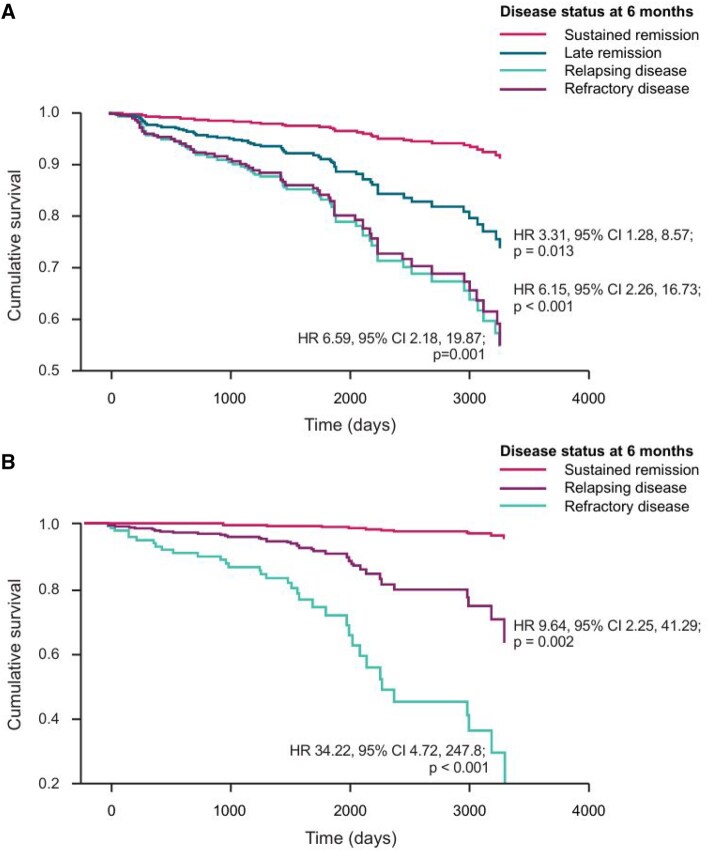
Cox proportional hazard curves showing the correlation between AAV control and long-term mortality **(A)** and ESKD **(B)** [6]. Figure reproduced from Gopaluni *et al*. 2019 [6] with permission from the American College of Rheumatology; permission conveyed through Copyright Clearance Center, Inc. Sustained remission was defined as remission at 3 months and sustained at 6 months. Late remission was defined as remission after 3 months and before 6 months. Relapsing disease was defined as remission at 3 months but relapse by 6 months. Refractory disease was defined as no remission by 6 months. AAV, ANCA-associated vasculitis; ESKD, end-stage kidney disease; HR, hazard ratio

Pooled data from four European Vasculitis Society (EUVAS) studies (*N* = 354) show that the risk of mortality and end-stage kidney disease (ESKD) was lower in patients achieving rapid and sustained AAV remission (remission by 3 months, sustained to 6 months) compared with patients with late remission (remission after 3 months and before 6 months), AAV relapse (remission by 3 months with relapse before 6 months) and/or refractory disease (no remission by 6 months) (Fig. 1A and B) [6]. This suggests that greater efforts are required to reduce the time to remission and prevent relapse.

AAV treatment guidelines typically recommend treating the majority of MPA/GPA patients with induction therapy based on RTX in preference to CYC [3–5]. This includes children/adolescents, premenopausal women and men concerned about their fertility, frail older adults, patients for whom GC-sparing is especially important and patients with relapsing disease and/or PR3-ANCA positivity [7]. However, RTX-based induction may be complicated by a relatively slow response to RTX [8] and a need for higher GC doses in patients treated with RTX *vs* CYC [8]. Furthermore, there is little evidence for the benefits of RTX in patients with low baseline estimated glomerular filtration rates (eGFR) [9].

A retrospective study in 129 patients with newly diagnosed or relapsing AAV (58.1% of whom had rapidly progressing glomerulonephritis [RPGN]) found that patients receiving a combination of RTX plus CYC rapidly achieved AAV remission while receiving lower doses of GC, with 84% of this difficult-to-treat population achieving remission by 5 months [10]. Consistent with these data, KDIGO treatment guidelines recommend using RTX-CYC combination therapy in patients with AAV and RPGN [5]. However, there is a paucity of data evaluating the safety and efficacy of combination therapy in other patient populations, and (with the exception of the RITUXVAS trial, which compared RTX-CYC *vs* CYC in patients receiving long-term CYC infusions [11]), there are no studies comparing RTX-CYC with either RTX or CYC alone. This suggests a need for head-to-head studies evaluating whether the addition of CYC to RTX in patients receiving standard treatment for AAV can help reduce the time to AAV remission and extend the duration of remission, while enabling more rapid GC tapering.

### Reducing GC exposure

Optimal GC dosing schedules for patients with new-onset AAV have been evaluated in the PEXIVAS trial [12] and the LoVAS trial [13]. The 7-year PEXIVAS trial found that reduced-dose oral GC was non-inferior to standard-dose GC in patients with severe AAV and kidney impairment (eGFR < 50 ml/min/1.73 m^2^), with the primary end point (death from any cause or ESKD) occurring in 92/330 patients (27.9%) in the low-dose group compared with 83/325 (25.5%) in the standard-dose group (absolute risk difference 2.3 percentage points [90% CI −3.4, 8.0]) [12]. Similarly, the LoVAS trial reported that the proportion of patients with newly diagnosed AAV achieving disease remission at 6 months was 49/69 (71.0%) in patients receiving reduced-dose GC plus RTX compared with 45/65 (69.2%) in the high-dose GC plus RTX group, with the low-dose regimen demonstrating noninferiority to the high-dose regimen [13]. As expected, the incidence of serious infections was significantly lower in the low-dose group in both trials. Based on results from the PEXIVAS trial [12], AAV treatment guidelines recommend rapidly tapering GC doses from approximately 60 mg/day to 5 mg/day within 4-5 months [3–5].

Avacopan was developed as a GC-sparing anti-vasculitis treatment [14–16]. This raises the question of how to manage concomitant GC alongside avacopan, which patients are likely to benefit most from avacopan treatment, and whether avacopan can be used to fully replace GC during induction therapy. The potential use of avacopan as an anti-vasculitis monotherapy has not yet been investigated.

### Assessing and/or predicting response to treatment

Response to AAV treatment is typically assessed using the Birmingham Vasculitis Activity Score (BVAS), with remission defined as BVAS = 0 [17]. The limitations of BVAS are that it is semi-objective, relies on clinical opinion, and only measures disease activity in the previous 28 days. Consequently, the definition of remission is sometimes expanded to include BVAS = 0 in patients receiving prednisolone doses ≤10 mg/day or complete GC withdrawal for a period of at least 1 month. Serological markers, such as CRP, ESR and ANCA, play a limited role in defining remission.

ANCA levels are often used to help diagnose AAV, identify AAV subtype and may be used as reliable markers for predicting relapse risk. A Cox-proportional hazard model in patients completing RTX-based therapy found that the risk of relapse was significantly higher in patients with ANCA positivity (hazard ratio [HR] 2.73 [95% CI 1.56, 4.8]; *P* < 0.001), a history of relapsing *vs* new-onset or refractory disease (HR 1.77 [0.94, 3.3]; *P* = 0.079) and in patients with AAV-related manifestations affecting the ears, nose and throat (ENT) (HR 2.35 [0.91, 6.1]; *P* = 0.077) [18].

**Figure 2.**
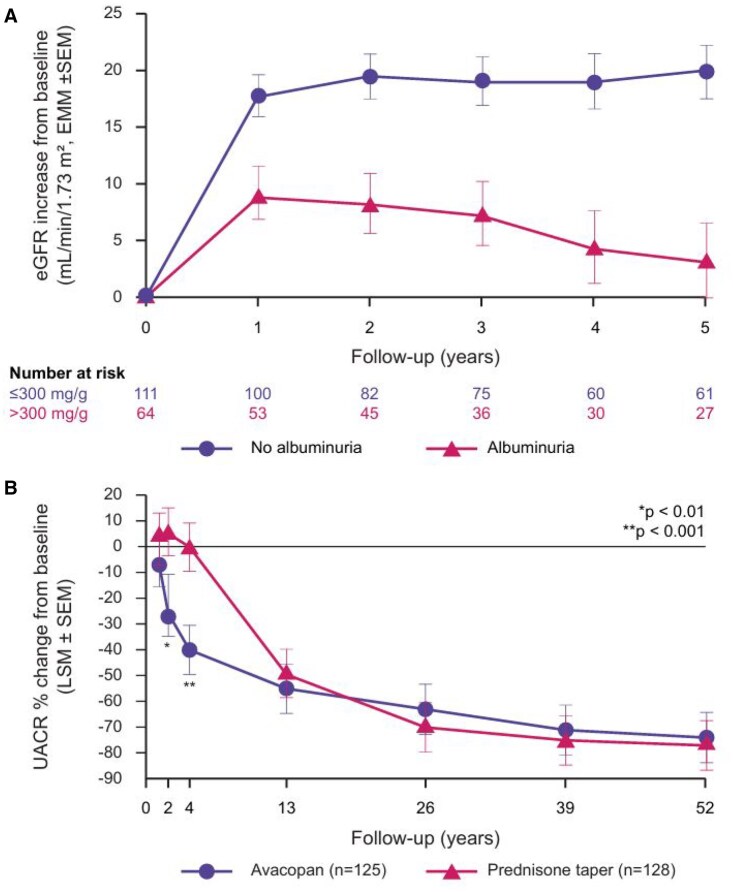
Impact of proteinuria on kidney function in AAV patients receiving induction/maintenance therapy [19] (**A**) and avacopan vs prednisone taper [20] (**B**). Panel **A** shows changes from baseline to 5 years in eGFR in patients with ANCA-associated GN with and without proteinuria receiving induction and/or maintenance therapy. Panel **B** shows changes in UACR from baseline to 52 weeks in patients with AAV treated with avacopan or prednisone taper in the ADVOCATE trial [20]. **A** is reproduced from Chalkia *et al*. [19] under the terms of the Creative Commons Attribution License (https://creativecommons.org/licenses/by/4.0/). **B** is reproduced from Geetha *et al*. [20] with permission from Oxford University Press; permission conveyed through Copyright Clearance Center, Inc. Albuminuria was defined as UACR >300 mg/g at 6 months. AAV, anti-neutrophil cytoplasmic associated vasculitis; eGFR, estimated glomerular filtration rate; EMM, estimated marginal means; GN, glomerulonephritis; LSM, least squares mean; SEM, standard error of mean; UACR, urinary albumin to creatinine ratio

A key aim for AAV induction therapy is the early recovery and/or stabilization of kidney function, monitored via changes in eGFR and serum creatinine. Although 50% of patients have persistent urinary abnormalities after 3–6 months of treatment, proteinuria and hematuria have largely been ignored as markers for kidney function. A retrospective, single-centre study in 218 patients with biopsy-proven ANCA-associated GN treated with induction therapy and/or maintenance therapy for up to 5 years found that patients without proteinuria at 6 months (defined as urinary albumin-to-creatinine ratio [UACR] ≤300 mg/g) had significantly greater increases in eGFR per year than patients with proteinuria (−12.5 ml/min/1.73 m^2^ [95% CI 9.1, 15.8]; *P* < 0.001) (Fig. 2A) [19]. This suggests that proteinuria thresholds should be considered when predicting response to treatment in patients with AAV and kidney impairment.

Data from the phase 2 CLEAR trial [15] and the phase 3 ADVOCATE trial [14, 20] found that proteinuria improved approximately three times faster in patients treated with avacopan *vs* prednisone taper. In the ADVOCATE trial, baseline geometric mean UACR in the avacopan group (*n* = 125) and the prednisone taper group (*n* = 128) was 433 mg/g (20–6461 mg/g) and 312 mg/g (11–5367 mg/g), respectively [20]. Significant UACR reductions from baseline occurred as early as 2 weeks with avacopan compared with ∼10–12 weeks with prednisone taper. Furthermore, the LSM reduction in UACR from baseline was significantly greater in the avacopan group *vs* the prednisone taper group at week 2 (difference −29% [95% CI −45%, −9%]), with the difference continuing to grow at week 4 (−40% [−53%, −22%]) before narrowing at week 13 (−12% [95% CI −32%, 13%]) (Fig. 2B). Rapid improvements in proteinuria in the avacopan group were reflected by earlier changes in markers of macrophage activation (e.g. monocyte chemoattractant protein-1) and likely account for the greater improvements in mean eGFR with avacopan (+7.6 ml/min/1.73 m^2^) *vs* prednisone taper (+4.6 ml/min/1.73 m^2^) at week 52. This suggests UACR might be a useful early indicator for improving kidney function in patients with AAV.

## Recommendations for avacopan use in the clinic

Primary endpoints from the phase 3 ADVOCATE trial show that avacopan (*n* = 166) was noninferior to prednisone taper (*n* = 165) for AAV remission (defined as BVAS = 0 and no receipt of GCs for 4 weeks) at week 26 (*P* < 0.001 for non-inferiority), and was superior to prednisone taper for sustained remission at week 52 (defined as remission [BVAS = 0] at weeks 26 and 52 and no receipt of GCs for 4 weeks before week 52) (*P* = 0.007 for superiority) [14]. Secondary endpoints show that avacopan reduced the absolute risk of AAV relapse over 52 weeks by 46% compared with prednisone taper (HR 0.46 [95% CI 0.25, 0.84]) while reducing GC exposure by >50% (mean GC dose 1676 mg with avacopan *vs* 3847 mg with prednisone taper) [14]. This may suggest a particular benefit for avacopan in patients needing to reduce their GC exposure due to GC-related adverse events (AEs) (e.g. diabetes mellitus, infections and osteoporosis) and implies that avacopan might be useful in patients with refractory AAV.

Subgroup analyses of ADVOCATE trial data demonstrate benefits for avacopan across diverse patient populations [21], including those with new-onset and relapsing disease [22], older patients [23] and in patients with manifestations related to the lung [24], ENT [24], chronic inflammation [25], nervous system [25], mucous membrane/eyes [25] and skin [25]. Furthermore, patients treated with avacopan had greater gains from baseline to week 52 in eGFR than patients receiving prednisone taper (treatment difference 3.2 ml/min/1.73 m^2^ [95% CI 0.3, 6.1]; *P* = 0.029) [14], with more pronounced gains in the subgroup of patients with baseline eGFR ≤20 ml/min/1.73 m^2^ (*n* = 50) (treatment difference 8.4 ml/min/1.73 m^2^ [95% CI 2.9, 13.8]; *P* = 0.003) [26]. This suggests a particular benefit for avacopan in patients with acute kidney injury.

Avacopan is administered as easy-to-swallow capsules at a fixed dose of 30 mg twice daily [27]. Evidence from the ADVOCATE trial [14] suggests avacopan should be used as part of induction therapy based on RTX or CYC with pre-existing prednisone tapered to zero after 4 weeks [27]. Although not confirmed, there is limited evidence to suggest that some patients with less severe manifestations who have not yet started GC might benefit from avacopan-based therapy without GC [14, 28, 29].

According to EULAR treatment guidelines [3], avacopan (when routinely used as part of induction therapy) should be continued for 1 year. This recommendation is based on results from the 52-week ADVOCATE trial, which demonstrate a lower risk of relapse for avacopan *vs* prednisone taper throughout the trial, with the greatest benefit occurring 26–52 weeks after treatment initiation and superiority for avacopan *vs* prednisone taper for sustained remission at 52 weeks [14]. However, emerging data suggest that some patients, in particular those with refractory disease and/or an intolerance or inadequate response to RTX, might benefit from extended avacopan treatment beyond 1 year [30, 31]. Results from the ongoing AvacoStar trial (NCT05897684) will provide data on the long-term safety of avacopan.

The most commonly reported adverse reactions in patients receiving avacopan are nausea (23.5%), headache (20.5%), neutropenia (18.7%), upper respiratory tract infections (14.5%) and diarrhea, vomiting and nasopharyngitis (15.1% each) [27], and the most commonly observed serious AEs are liver function abnormalities (5.4%) and pneumonia (4.8%) [27]. All patients receiving avacopan should be monitored for changes in liver enzymes and white blood cell counts [27].

## Avacopan use in the real world

**Figure 3.**
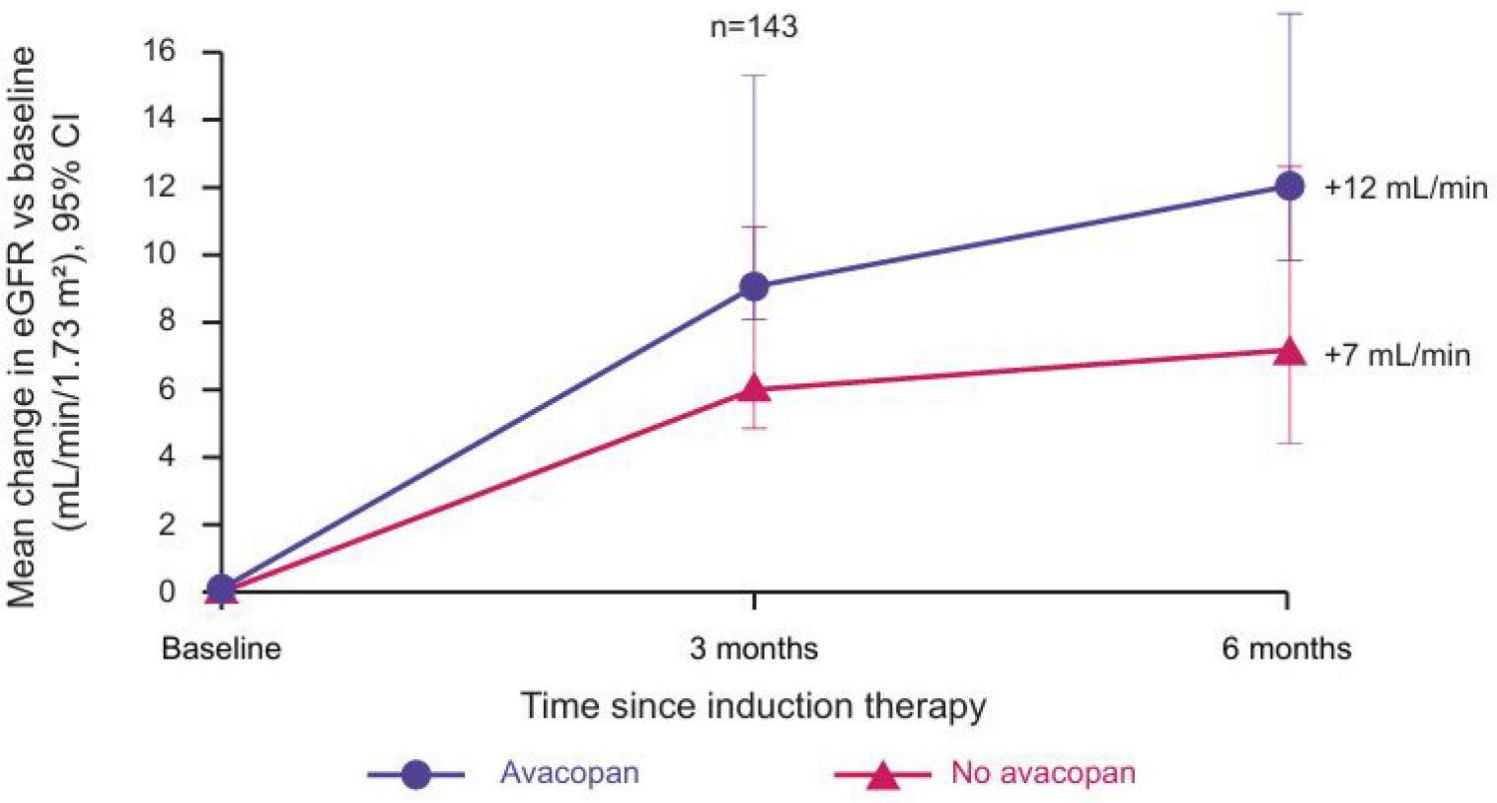
Mean change in eGFR among 143 patients with AAV and acute kidney injury treated with vs without avacopan-based therapy (UK Registry data) [32]. eGFR, estimated glomerular filtration rate

UK Registry data from 143 patients with AAV and acute kidney injury show higher rates of kidney function recovery in patients receiving avacopan-based therapy *vs* non-avacopan-based therapy (Fig. 3) [32]. The change in eGFR from baseline to 6 months was 5 ml/min/1.73 m^2^ greater in the avacopan group *vs* the non-avacopan group in the real-world study compared with 5.8 ml/min/1.73 m^2^ in patients with baseline eGFR ≤20 ml/min/1.73 m^2^ in the ADVOCATE trial [26]. This suggests the effects of avacopan on eGFR in clinical trials directly translate to the real world.

Subgroup analyses of ADVOCATE trial data demonstrate benefits for avacopan across a wide range of AAV patient populations with and without kidney manifestations [21–26]. Although patients with RPGN and eGFR <15 ml/min/1.73 m^2^ were excluded from the ADVOCATE trial [14], evidence from a real-world case study in three patients with RPGN requiring dialysis at baseline found that treatment with avacopan, RTX and/or CYC and a rapid GC taper enabled all three patients to recover sufficient kidney function to discontinue dialysis [33]. Similarly, the ADVOCATE trial excluded patients with pulmonary hemorrhage [14]. However, a case series of eight patients with GPA/MPA and hypoxic pulmonary hemorrhage (four of which required mechanical ventilation in intensive care) found that all patients achieved complete resolution of lung symptoms after receiving avacopan for a median of 10 days (range 2–40 days), with all eight patients achieving sustained remission and none requiring rehospitalization at a median of 6 months (range 2–13 months) [34]. Although limited, real-world evidence supports the use of avacopan in patients with AAV and RPGN or pulmonary hemorrhage.

### Case study 1

A female patient with osteoporosis in her 60s presented to hospital with a 6-month history of fever, weight loss, cough and sinusitis. Laboratory evaluations found ESR >100 mm/h, CRP 145 mg/l (0–6), PR3-ANCA 18 IU/ml (0–1.9), creatinine 63 µmol/l and no abnormal urinary values. A CT scan of her lungs showed bilateral consolidations involving the posterior and apical segment of the upper lobes and several pulmonary nodules. Additional tests ruled out malignancy and pulmonary infection and the patient was diagnosed with GPA with lung and ENT disease without renal involvement. The treatment-naïve patient was recruited into the ADVOCATE trial where she received avacopan 30 mg BID and RTX 375 mg/m^2^/week for 4 weeks. Avacopan was continued for 1 year before transitioning to RTX-based maintenance therapy for a further 2 years. Remission (BVAS = 0) was achieved by week 5, CRP levels improved and ANCA levels decreased to zero after ∼9 months. Sequential CT scans showed progressive resolution of pneumonic consolidation over the next 2 years, leaving some bilateral nodules and scaring. This case study supports the use of avacopan and RTX in patients with GPA, pulmonary impairment and contraindications for GC.

### Case study 2

A male patient in his 50s presented to hospital with cough, vision loss, CRP 205 mg/l, PR3-ANCA 102 IU/ml, hemoglobin 97 g/l and no signs of hematuria or proteinuria. CT chest images showed consolidation with hemorrhage and large nodules, malignancy and infection were excluded, and the patient was diagnosed with GPA and optic neuritis. Aggressive induction therapy was prescribed to reduce the risk of further vision loss. This included intravenous (IV) methylprednisolone (1.5 g total), high-dose oral GC, plasma exchange, IV CYC and IV RTX (1 g), which was discontinued following a severe reaction. The patient achieved rapid reductions in PR3-ANCA but CRP levels continued to fluctuate, and the patient was hospitalized seven times for serious lung infections (including *Staphylococcus aureas*, *aspergillosis* and COVID-19 infections), lung vasculitis and/or atrial fibrillation. Infections were attributed to the extent of GPA lung disease and the use of high-dose steroids and were treated with antibiotics. The patient developed steroid-induced diabetes (glycated hemoglobin [HbA1c] 71%), gained 11 kg in weight and suffered a relapse at month 7 characterized by rapidly increasing PR3-ANCA, hemoptysis, worsening lung nodules/cavities and an additional lung infection. Treatment of refractory AAV using CYC, intravenous immunoglobulin (IVIG), and avacopan with GC tapering led to reductions in ANCA levels and remission at 3 months. Eighteen months later, the patient was still in remission with CRP <4 mg/l, PR3-ANCA 2.6 IU/ml, no RTX or azathioprine maintenance therapy, full prednisolone withdrawal and improving HbA1c levels (33%). A follow-up CT scan showed one small nodule and scarring. This case study supports the use of avacopan in patients with RTX intolerance and relapsing GPA with structural lung disease complicated by AAV relapse and a slowed treatment response due to severe infections and the use of high-dose GC.

## Emerging treatments for AAV

A number of potential therapies are being investigated for the treatment of AAV. These include B cell depletion therapies (e.g. obinutuzumab, the anti-CD38 therapy daratumumab and CAR-T therapy), therapies targeting B cell cytokines (e.g. the monoclonal anti-BLγS antibodies belimumab and APRIL) and therapies targeting B cell activation (e.g. abatacept and ustekinumab) [35, 36]. Belimumab and abatacept were recently shown to be ineffective in patients with AAV [35]; consequently, development of these agents has been discontinued. In addition to the B cell targeted therapies, development is ongoing for other alternative complement inhibitors and there is some interest in neutrophil- and fibrosis-targeted therapies.

## Summary and conclusions

Achieving early and sustained remission [37] while reducing patient exposure to GC [12, 13] remain key goals for the effective management of patients with AAV. The ADVOCATE trial has consistently shown that avacopan enables sustained remission, considerable reductions in GC exposure and improved recovery of kidney function in a wide range of patients with MPA/GPA with and without kidney manifestations [23]. Although avacopan might be particularly beneficial in certain patient groups (e.g. those with low eGFR [24], refractory disease [14] and/or GC toxicity [14]), its use should be considered in all patients requiring induction therapy [38]. Limited but increasing evidence from real-world studies supports the use of avacopan in patients with RPGN [33] and AAV-related pulmonary hemorrhage [34], and suggests that some patients (particularly those with refractory disease) might benefit from longer-term avacopan exposure beyond 12 months [30, 31].
